# Reduction of Cardiac Autonomic Modulation and Increased Sympathetic Activity by Heart Rate Variability in Patients With Long COVID

**DOI:** 10.3389/fcvm.2022.862001

**Published:** 2022-04-29

**Authors:** Karina Carvalho Marques, Camilla Costa Silva, Steffany da Silva Trindade, Márcio Clementino de Souza Santos, Rodrigo Santiago Barbosa Rocha, Pedro Fernando da Costa Vasconcelos, Juarez Antônio Simões Quaresma, Luiz Fábio Magno Falcão

**Affiliations:** ^1^Postgraduate Program in Parasitic Biology in the Amazon, Laboratory of Infectious and Cardiopulmonary Diseases, Long COVID Program, Centre for Biological and Health Sciences, Pará State University, Belém, Brazil; ^2^Laboratory of Infectious and Cardiopulmonary Diseases, Long COVID Program, Centre for Biological and Health Sciences, Pará State University, Belém, Brazil; ^3^Department of Human Movement Sciences, Centre for Biological and Health Sciences, Pará State University, Belém, Brazil; ^4^Centre for Biological and Health Sciences, Pará State University, Belém, Brazil

**Keywords:** autonomic nervous system, coronavirus infection, long COVID, heart rate, heart rate variability

## Abstract

Although several clinical manifestations of persistent long coronavirus disease (COVID-19) have been documented, their effects on the cardiovascular and autonomic nervous system over the long term remain unclear. Thus, we examined the presence of alterations in cardiac autonomic functioning in individuals with long-term manifestations. The study was conducted from October 2020 to May 2021, and an autonomic assessment was performed to collect heart rate data for the heart rate variability (HRV) analysis. The study participants were divided into the long COVID clinical group, the intragroup, which included patients who were hospitalized, and those who were not hospitalized and were symptomatic for different periods (≤3, >3, ≤6, and >6 months), with and without dyspnoea. The control group, the intergroup, comprised of COVID-free individuals. Our results demonstrated that the long COVID clinical group showed reduced HRV compared with the COVID-19-uninfected control group. Patients aged 23–59 years developed COVID symptoms within 30 days after infection, whose diagnosis was confirmed by serologic or reverse-transcription polymerase chain reaction (swab) tests, were included in the study. A total of 155 patients with long COVID [95 women (61.29%), mean age 43.88 ± 10.88 years and 60 men (38.71%), mean age 43.93 ± 10.11 years] and 94 controls [61 women (64.89%), mean age 40.83 ± 6.31 and 33 men (35.11%), mean age 40.69 ± 6.35 years] were included. The intragroup and intergroup comparisons revealed a reduction in global HRV, increased sympathetic modulation influence, and a decrease in parasympathetic modulation in long COVID. The intragroup showed normal sympathovagal balance, while the intergroup showed reduced sympathovagal balance. Our findings indicate that long COVID leads to sympathetic excitation influence and parasympathetic reduction. The excitation can increase the heart rate and blood pressure and predispose to cardiovascular complications. Short-term HRV analysis showed good reproducibility to verify the cardiac autonomic involvement.

## Introduction

Coronavirus disease (COVID-19), caused by severe acute respiratory syndrome coronavirus-2 (SARS-CoV-2), manifests numerous clinical symptoms, ranging from mild to severe (1). In Brazil, 21,478,546 confirmed cases, 598,152 accumulated deaths, and 20,462,345 recovered cases were reported as of October 4, 2021. In the state of Pará, 591,872 COVID-19 cases and 16,667 deaths were registered (2).

Some patients who do recover present with symptoms that persist longer than 3–4 weeks. According to Sher (3), this post-COVID condition may be called “post-COVID syndrome,” “long COVID,” or “post-acute COVID-19.” The persistent symptoms of patients with long-term COVID-19 include dyspnoea, fatigue, myalgia, and joint pain (3, 4). The cardiovascular effects of prolonged COVID-19 are still under debate but they may include the lack of clinical symptoms, biomarker (high-sensitivity cardiac troponin I) abnormalities, or an increased risk of myocarditis. Coronavirus infection the potential to affect the cardiovascular system. SARS-CoV-2 is not considered a cardiotropic virus, although the virus causes non-specific cytokine-mediated cardiotoxicity (5, 6). Long COVID is found to be associated with autonomic dysfunction due to neurotropism because the systemic inflammatory state can occur acutely or chronically for up to 1 year (7). Dysautonomia can thus be assessed based on heart rate variability (HRV). A reduction in HRV is a predictor of cerebrovascular and cardiovascular events and an indicator of the risk of death (8). HRV as a non-invasive index of autonomic control may reflect both sympathetic and parasympathetic effects. The HRV indicates the variations in the duration of cardiac cycles and the RR intervals. HRV can be analyzed using linear algorithms for the time and frequency domains and also by non-linear analyses (9, 10).

Changes in cardiac autonomic modulation can occur in patients with COVID-19 and, through HRV, detect autonomic dysregulation. Patients with COVID-19 with low HRV are indicated for intensive care unit admission in the first week after hospitalization, regardless of age and chronic heart disease status (11). In this context, the aim of this study was to investigate the autonomic changes in the heart among patients with long COVID.

## Methods

### Study Design and Ethics

This observational, analytical, controlled, quantitative, and descriptive study was approved by the Research Ethics Committee of the Pará State University (approval number 4.252.664). Participants consented to be included in the study by signing an informed consent form; the study was performed following the Strengthening the Reporting of Observational Studies in Epidemiology guidelines for observational studies and in accordance with the principles of the Declaration of Helsinki.

The patients were followed up at the laboratory of infectious and cardiopulmonary diseases at UEPA. The study participants were divided into a long COVID clinical group (intragroup), which comprised both patients who were hospitalized and those who were not hospitalized but were symptomatic for ≤3, >3, ≤6, and >6 months, with and without dyspnoea, as well as a control group (intergroup), which comprised COVID-free individuals. As it was a cardiopulmonary program, patients with dyspnoea and respiratory symptoms such as shortness of breath were required to adhere to the program. The following patients were included in the study: those aged between 23 and 59 years, those who underwent assessment 30 days after diagnostic confirmation and onset of COVID-19 symptoms, and those whose diagnosis was confirmed by reverse-transcriptase polymerase chain reaction (PCR) or serology tests to identify the type of antibodies [immunoglobulin (Ig) M and/or IgG]. Patients who used medication that altered the HRV (such as beta-blockers, beta-mimetics, and theophylline), those who developed chronic obstructive pulmonary disease, those who had persistent lung changes, those who showed persistent desaturation, those with anemia, and those who used pacemakers were excluded. Patients with long COVID underwent regular medical examinations.

From October 2020 to May 2021, 4,100 patients with complaints of long-term symptoms (like fatigue, breathlessness, cough, joint pain, chest pain, muscle aches, and headaches) that could not be attributed to any other cause were enrolled in the clinic's database. However, only 155 patients met the inclusion criteria.

### Assessment of HRV

Study participants in both groups were instructed to refrain from consuming caffeine or caffeine derivatives, smoking, and eating heavy meals at least 24 h prior to the test. In preparation for the examination, the volunteers rested for 15 min, while the patients were placed in a supine position for 10 min to measure the heart rate (HR). The patients were instructed to avoid talking or moving to prevent interference during the test. The environment temperature was maintained between 22 and 24°C, while the air humidity was maintained between 40 and 60%. The temperature and relative humidity were measured using a thermo-hygrometer (São Paulo, Brazil).

The Heart Rate (HR) was recorded using a Polar® RS800CX (Kempele, Finland) that captured the R wave on the electrocardiogram with a sampling rate of 500 Hz. The temporal distance between two consecutive peaks of the R wave was considered the iRR (the fluctuations in the intervals between consecutive heartbeats). The data series displayed on the monitor were exported using the Polar Pro Trainer 5 software (Polar Electro Ou, Finland). Subsequently, linear and nonlinear analyses were performed using the Kubios HRV version 3.1 software (Kuopio, Finland). Variability analysis was performed within a short period of time (12) to visually check the distribution of the iRRs for erroneous and absent R waves to determine the stretch with greater stability within 5 min for 256 consecutive beats. Then, the data collected during the first 30 s and final 30 s were discarded (10).

### Linear Analysis

In the linear analysis of the HRV in the time domain, the following indices were included: mean iRRs, standard deviation of all normal RR intervals (SDNN), and the square root of the mean square of the differences between adjacent normal iRRs within an interval (RMSSD). SDNN indicates the sympathetic nervous system (SNS) and parasympathetic nervous system (PNS) activities, whereas RMSSD indicates the PNS (13) activity. To obtain the indices in the frequency domain, a fast Fourier transform analysis was performed and showed the components with high frequency (HF = 0.15–0.4 Hz), low frequency (LF = 0.04–0.15 Hz), and very low frequency (VLF = 0.003–0.04 Hz), as indicated in equations 1 and 2 (13, 14).

The three frequency bands (HF, LF, and VLF) were expressed in powers (ms^2^) and in normalized units (n.u), which is the relative power of the LF or HF band after subtracting the VLF power from the total power. The LF/HF ratio and LF and HF bands were also obtained (15). LF and HF in normalized units represent the balance between the two autonomic nervous systems. LF demonstrates indirect sympathetic activity, while HF demonstrates the parasympathetic influence. The LF/HF ratio was reported as a marker of sympathovagal balance (10).


(1) $$\begin{array}{r} HF\;\left(n.u\right)=100\;x\;\frac{\text{HF}}{\left(\text{full power}-\text{VLF}\right)} \end{array}$$

(2) $$\begin{array}{r} LF\;\left(n.u\right)=100\;x\;\frac{\text{LF}}{\left(\text{full power}-\text{VLF}\right)}. \end{array}$$

### Nonlinear Analysis

For the nonlinear analysis, geometric methods, such as the Lorenz plot or Poincaré plot, were used to obtain the HRV measurements. These were performed by measuring the dispersions of interval RRs which analyse the HRV quantitatively, by calculating the beat-to-beat standard deviation (SD). With this, the short term changes in RRs in the PNS index of the sinoatrial node control (SD1) and the long-term standard deviation of continuous iRRs (SD2), which are influenced by the PNS and SNS were measured, and the relationship between the short- term and the long-term intervals was defined as SD1/SD2 (15, 16).

In addition, other nonlinear methods based on approximate entropy (ApEn) show the degree of irregularity and complexity of the signal as the iRRs and the complexity increase (16, 17). Meanwhile, simple entropy (SampEn) shows the regularity of the selected iRR series; higher values indicate a healthy condition, while lower values indicate heart failure (10, 18).

### Statistical Analysis

The information collected was stored in MS Excel 2010™ (Washington, United States) and analyzed using GraphPad Prism version 5.0™ (San Diego, United States). D'Agostino's test was used to assess the normality of the distribution to compare the measured values between the different study groups. The Student's *t-*test was used for variables with a normal distribution, whereas the Mann–Whitney *U* test was used for non-normally distributed variables. For dichotomous or nominal variables, Fisher's exact test was used. A two-tailed *P*-value of <0.05 was considered significant.

## Results

### General Characteristics of the Study Patients

**Figure 1:**
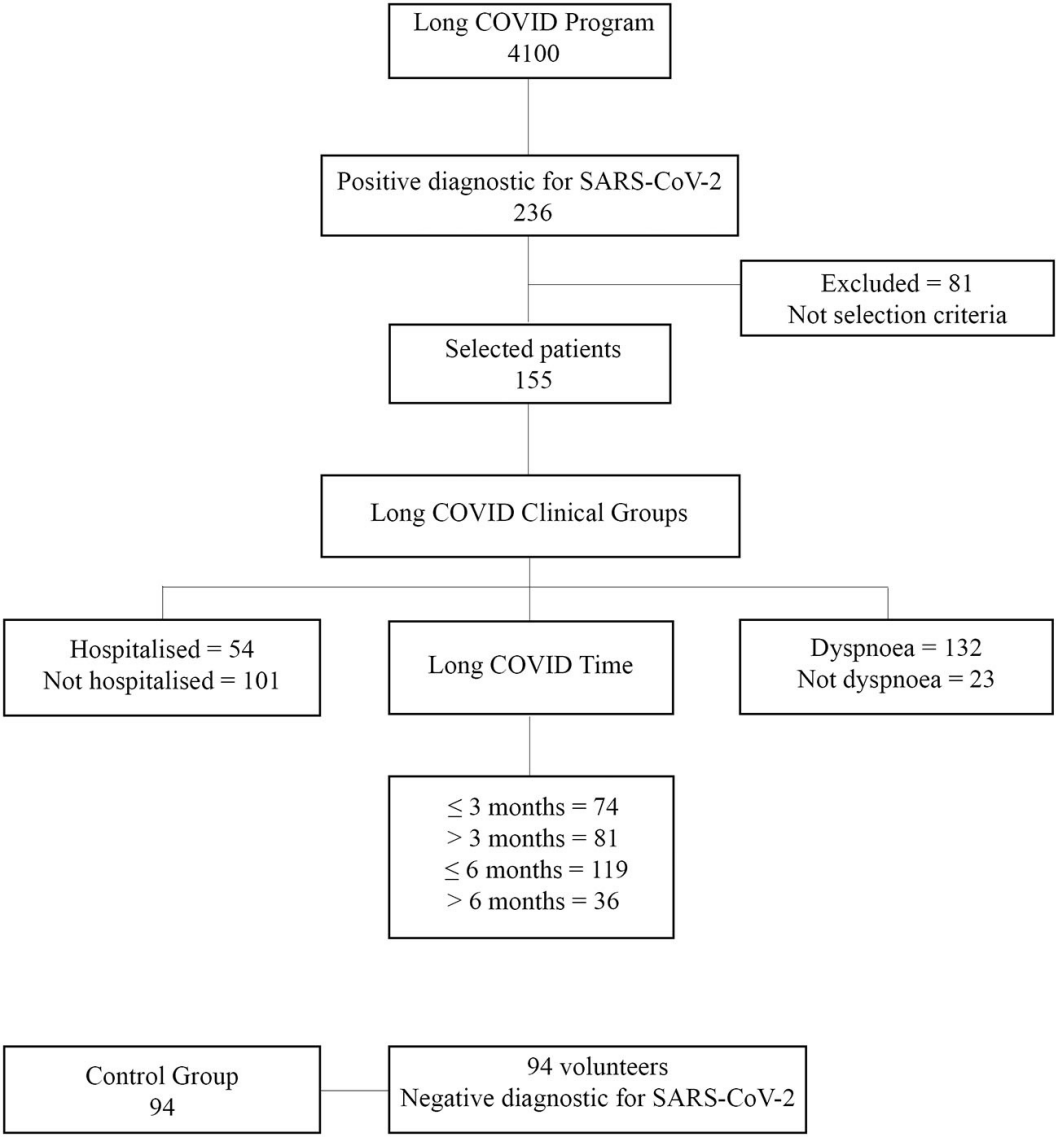
Flowchart of selection and recruitment of patients with long COVID. SARS-CoV-2, severe acute respiratory syndrome coronavirus-2; COVID, coronavirus disease.

After screening 236 patients, 155 were included in the study, as shown in Figure 1. The demographic characteristics and comorbidities of the 155 patients with long COVID symptoms are listed in Table 1. Most participants were women with a mean age of over 40 years; dyspnoea was one of the most prevalent symptoms of long COVID, accounting for more than 30% of hospitalized cases.

**Table 1:** Demographic characteristics and comorbidities of the study population and symptoms of long COVID.

**Variables**	**Patients (*n* = 155)**	**Group control (*n* = 94)**
Female, No (%)	95 (61.29%)	61 (64.89%)
Male, No (%)	60 (38.71%)	33 (35.11%)
Age (years), mean ± SD	43.88 ± 10.03	40.69 ± 6.35
Height (cm), mean ± SD	1.63 ± 0.08	1.64 ± 0.08
Weight (kg), mean ± SD	79.95 ± 17.84	73.73 ± 15.43
BMI, mean ± SD	30.06 ± 7.31	27.04 ± 4.30
Smoker (No, %)	3 (1.93%)	N/A
Former smoker (No, %)	28 (18.06%)	94 (100%)
**Long COVID symptoms (No, %)**		
Dyspnoea, No (%)	132 (85.16%)	N/A
Chest pain, No (%)	93 (60%)	N/A
Muscle weakness, No (%)	112 (72.25%)	N/A
Fatigue, No (%)	118 (76.12%)	N/A
Myalgia, No (%)	103 (66.45%)	N/A
Insomnia, No (%)	87 (56.12%)	N/A
Lower members Oedema, No (%)	58 (37.41%)	N/A
**Comorbidities (No, %)**		N/A
Asthma (No, %)	24 (15.48%)	N/A
DM (No, %)	13 (8.38%)	N/A
SAH (No, %)	34 (21.93%)	N/A
Obesity (No, %)	72 (46.45%)	28 (29.78%)
**Hospital admission (** ***n*** **, %)**	54 (34.83%)	
**Length of hospital stay, mean** **±SD**	17.25 ± 15.96	N/A
≤10 days, (*n*, %)	19 (35.18%)	N/A
>10 days, (*n*, %)	35 (64.82%)	N/A
**Long COVID period, mean** **±SD**		N/A
≤3 months, (*n*, %)	74 (47.74%)	N/A
>3 months, (*n*, %)	81 (52.26%)	N/A
≤6 months, (*n*, %)	119 (76.77%)	N/A
>6 months, (*n*, %)	36 (23.23%)	N/A
**Dyspnoea**	132 (85.16%)	N/A
**Not dyspnoea**	23 (14.84%)	N/A

*BMI, body mass index; DM, diabetes mellitus; SAH, systemic arterial hypertension; SD, standard deviation*.

### Intragroup Comparison

Intragroup comparisons were performed between the long COVID clinical groups. The mean age of the groups was >40 years, with >40% of them admitted to the hospital for 3 months or longer, while 80% experienced dyspnoea. The demographic characteristics, comorbidities, and symptoms of the long COVID groups are shown in Table 2.

**Table 2:** Demographic characteristics, comorbidities, and symptoms of the study population considering the long COVID clinical group.

**Variables**	**Hospitalised (*n* = 54) No, (%)**	**Not hospitalised (*n* = 101) No, (%)**	***P*-Value**	**≤3 months (*n* = 74) No, (%)**	**>3 months (*n* = 81) No, (%)**	***P*-Value**	**≤6 months (*n* = 119) No, (%)**	**>6 months (*n* = 36) No, (%)**	***P*-Value**	**Dyspnoea (*n* = 132) No, (%)**	**Not dyspnoea (*n* = 23) No, (%)**	***P*-Value**
**Sex**												
Female	24 (44.44%)	71 (70.29%)	^*^0.002	38 (51.35%)	57 (70.37%)	^*^0.020	67 (56.30%)	28 (77.77%)	^*^0.020	83 (62.87%)	12 (52.17%)	0.359
Male	30 (55.56%)	30 (29.71%)		36 (48.65%)	24 (29.63%)		52 (43.70%)	8 (22.23%)		49 (37.13%)	11 (47.83%)	
Mean age (years)	44.27 ± 9.22	43.67 ± 10.47	0.837	42.17 ± 10.77	45.44 ± 9.09	0.074	43.78 ± 10.61	44.19 ± 7.90	0.940	43.43 ± 9.56	46.47 ± 12.30	0.180
Stature	1.64 ± 0.09	1.63 ± 0.08	0.441	1.64 ± 0.09	1.62 ± 0.08	0.221	1.63 ± 0.09	1.63 ± 0.08	0.739	1.63 ± 0.08	1.63 ± 0.09	0.938
Weight	86.80 ± 18.41	76.29 ± 16.49	^*^0.000	82.52 ± 19.21	77.60 ± 16.26	0.118	79.79 ± 17.80	80.49 ± 18.24	0.581	79.53 ± 17.78	82.35 ± 18.42	0.396
BMI	32.23 ± 7.11	28.90 ± 7.18	^*^0.005	30.70 ± 7.81	29.48 ± 6.82	0.361	29.94 ± 7.19	30.48 ± 7.80	0.684	29.89 ± 7.22	31.07 ±7.88	0.422
**Smoker**												
Yes	0	3 (2.97%)	0.314	1 (1.35%)	2 (2.46%)	1.00	3 (2.52%)	3 (8.33%)	0.138	2 (1.51%)	1 (4.34%)	0.381
Not	54 (100%)	98 (97.03%)		73 (98.65)	79 (97.54%)		116 (97.48%)	33 (91.67%)		130 (98.49%)	22 (95.66%)	
**Ex-smoker**												
Yes	12 (22.22%)	6 (5.94%)	^*^0.006	5 (6.75%)	12 (14.81%)	0.128	10 (8.40%)	7 (19.44%)	0.123	17 (12.87%)	3 (13.04%)	0.986
Not	42 (77.78%)	95 (94.06%)		69 (93.25%)	69 (85.19%)		109 (91.6%)	29 (80.56%)		115 (87.13%)	20 (86.96%)	
**Long COVID symptoms (** ***n*** **, %)**												
Dyspnoea	47 (87.03%)	85 (84.15%)	0.813	60 (81.08%)	72 (88.88%)	0.183	97 (81.51%)	35 (97.22%)	^*^0.016	132 (100%)	0	0
Chest pain	31 (57.40%)	62 (61.38%)	0.731	43 (58.10%)	50 (61.72%)	^*^0.041	68 (57.14%)	25 (69.44%)	^*^0.021	78 (59.09%)	15 (65.21%)	0.649
Fatigue	40 (74.07%)	78 (77.22%)	0.695	52 (70.27%)	66 (81.48%)	0.131	86 (72.26%)	32 (88.88%)	^*^0.045	103 (78.03%)	15 (65.21%)	0.192
Muscle weakness	46 (85.18%)	66 (65.34%)	^*^0.008	55 (74.32%)	57 (70.37%)	0.595	81 (68.06%)	31 (86.11%)	^*^0.035	96 (72.72%)	16 (69.56%)	0.802
Myalgia	40 (74.07%)	63 (62.37%)	0.157	52 (70.27%)	51 (62.96%)	0.395	78 (65.54%)	25 (69.44%)	0.130	86 (65.15%)	17 (73.91%)	0.480
Insomnia	36 (66.66%)	51 (50.49%)	0.062	42 (56.75%)	45 (55.55%)	1.00	67 (56.30%)	20 (55.55%)	1.00	75 (56.81%)	12 (52.17%)	0.820
Lower members Oedema	28 (51.85%)	30 (29.70%)	^*^0.008	26 (35.13%)	32 (39.50%)	0.620	40 (33.61%)	18 (50%)		48 (36.36%)	10 (43.47%)	0.641
**Comorbidities**												
Asthma	3 (5.55%)	21 (20.79%)	^*^0.011	8 (10.81%)	16 (19.75%)	0.181	20 (16.80%)	4 (11.11%)	0.450	20 (15.15%)	4 (17.39%)	0.756
DM	7 (12.96%)	6 (5.94%)	0.221	6 (8.10%)	7 (8.64%)	1.00	11 (9.24%)	2 (5.55%)	0.733	12 (9.09%)	1 (4.34%)	0.693
SAH	11 (20.37%)	23 (22.77%)	0.839	18 (24.32%)	16 (19.75%)	0.561	28 (23.52%)	6 (16.66%)	0.492	28 (21.21%)	6 (26.08%)	0.591
Obesity	33 (61.11%)	39 (38.61%)	^*^0.010	37 (50%)	35 (43.20%)	0.423	53 (44.53%)	19 (52.77%)	0.447	61 (46.21%)	11 (47.82%)	1.00
**Hospital admission (** ***n*** **, %)**												
Yes	54 (100%)	0	0	32 (43.24%)	22 (27.16%)	^*^0.043	40 (33.61%)	14 (38.88%)	0.690	47 (35.60%)	7 (30.43%)	0.081
Not	0	101 (100%)		42 (56.76%)	59 (72.83%)		79 (66.69%)	22 (61.12%)		85 (64.40%)	16 (69.57%)	
**Mean length of stay (days)**												
≤10 days (*n*, %)	19 (35.18%)	0	0	11 (34.37%)	8 (9.87%)	1.00	15 (12.60%)	4 (28.57%)	0.747	16 (34.04%)	3 (42.85%)	0.686
>10 days (*n*, %)	35 (63.22%)	0		21 (65.63%)	14 (90.13%)		25 (87.4%)	10 (71.43%)		31 (65.96%)	4 (57.15%)	

*BMI, body mass index; MMII, lower member; DM, diabetes mellitus; SAH, systemic arterial hypertension; SD, standard deviation*.

* *P significant value*.

Regarding the HRV data, patients who experienced symptoms for >3 and >6 months had higher HRV, those who experienced symptoms for ≤3 months had reduced parasympathetic modulation and increased sympathetic modulation influence of, and those who were hospitalized had a reduction in the sympathovagal balance (Table 3).

**Table 3:** Analysis of HRV considering the long COVID clinical group.

**Variables**	**Hospitalised (*n* = 54) Mean ±SD**	**Not hospitalised (*n* = 101) Mean ±SD**	***P*-Value**	**≤3 months (*n* = 74) Mean ±SD**	**>3 months (*n* = 81) Mean ±SD**	***P*-Value**	**≤6 months (*n* = 109) Mean ±SD**	**>6 months (*n* = 28) Mean ±SD**	***P*-Value**	**Dyspnoea (*n* = 132) Mean ±SD**	**Not dyspnoea (*n* = 23) Mean ±SD**	***P*-Value**
RR (Ms)	794.16 ± 132.22	822.28 ± 126.72	0.228	806.93 ± 129.25	847.24 ± 138.30	^*^0.002	806.93 ± 129.25	830.86 ± 127.96	^*^0.002	812.97 ± 131.03	809.69 ± 118.88	0.925
SDNN (Ms)	28.47 ± 28.46	42.58 ± 119.92	0.171	39.84 ± 111.68	46.83 ± 133.77	0.122	39.84 ± 111.68	30.46 ± 19.96	0.571	39.81 ± 106.10	25.35 ± 20.63	0.208
RMSSD (Ms)	31.48 ± 37.22	35.05 ± 30.59	0.132	33.61 ± 34.34	38.25 ± 35.68	^*^0.043	33.61 ± 34.34	34.45 ± 28.44	0.529	34.57 ± 33.17	29.42 ± 32.35	0.438
SD1 (Ms)	22.29 ± 26.36	24.82 ± 21.66	0.133	23.80 ± 24.32	27.09 ± 25.27	^*^0.042	23.80 ± 24.32	24.40 ± 20.14	0.523	24.48 ± 23.48	20.82 ± 22.83	0.432
SD2 (Ms)	32.88 ± 31.12	35.27 ± 21.96	0.175	34.27 ± 26.80	35.93 ± 23.32	0.210	34.27 ± 26.80	35.00 ± 20.69	0.435	35.47 ± 26.30	28.52 ± 19.25	0.157
SD1/SD2	0.61 ± 0.25	0.66 ± 0.22	0.193	0.65 ± 0.24	0.69 ± 0.24	^*^0.011	0.65 ± 0.24	0.64 ± 0.20	0.876	0.64 ± 0.23	0.67 ± 0.23	0.604
ApEn	1.12 ± 0.15	1.14 ± 0.12	0.613	1.14 ± 0.13	1.12 ± 0.13	^*^0.007	1.14 ± 0.13	1.11 ± 0.15	0.176	1.14 ± 0.13	1.09 ± 0.16	0.079
SampEn	1.58 ± 0.37	1.63 ± 0.32	0.560	1.62 ± 0.34	1.59 ± 0.32	0.150	1.62 ± 0.34	1.59 ± 0.34	0.659	1.62 ± 0.33	1.55 ± 0.39	0.569
LF (n.u)	54.39 ± 21.54	48.83 ± 16.71	0.073	51.18 ± 19.26	47.29 ± 18.33	^*^0.014	51.18 ± 19.26	49.39 ± 16.66	0.533	50.39 ± 19.02	52.94 ± 16.60	0.572
HF (n.u)	45.54 ± 21.52	51.04 ± 16.84	0.079	48.69 ± 19.23	52.60 ± 18.33	^*^0.014	48.69 ± 19.23	50.56 ± 16.65	0.519	49.50 ± 18.99	46.97 ± 16.58	0.586
LF/HF	6.33 ± 30.60	1.26 ± 1.15	^*^0.032	1.73 ± 1.59	4.22 ± 25.06	^*^0.024	1.56 ± 1.56	7.90 ±37.51	0.887	3.30 ± 19.64	1.46 ± 1.12	0.657

*SD, standard deviation; RR, average of RR intervals; N.u, normalized units; SDNN, standard deviation of all normal RR intervals; RMSSD, square root of the mean square of the differences between adjacent normal RR intervals in a time interval; SD1, rapid changes in RR intervals in parasympathetic nervous system index; SD2, long-term changes; SD1/SD2, short term ratio for long-term range variation; ApEn, approximate entropy, complexity, regularity of the RR interval series and signal complexity; SampEn, simple entropy, regularity of the RR interval series; LF, low-frequency components, ranging from 0.004 to 0.15 hertz; HF, high-frequency components, ranging from 0.15 to 0.004 hertz; LF/HF, low/high frequency components (normal range 1.5 to 2.0)*.

* *P significant value*.

### Intergroup Comparison

Major changes were observed in the intergroup comparison between the long COVID clinical groups and the control group. The clinical groups showed a reduction in global HRV (RR, SDNN, SD2, and SD1/SD2), increased the influence of sympathetic modulation (LF, LF/HF), decreased parasympathetic modulation (RMSSD, SD1, and HF), and decreased sympathovagal balance of the heart (LF/HF) in relation to the control group that did not manifest COVID-19. Data are shown in Tables 4, 5.

**Table 4:** Analysis of HRV duration of long COVID and dyspnoea in the hospitalization groups based on the control group.

**Variable**	**Hospitalised (*n*= 54) Mean ±DP**	**Control group (*n* = 94) Mean ±SD**	***P*-Value**	**Not hospitalised (*n*= 101) Mean ±SD**	**Control group (*n* = 94) Mean ±SD**	***P*-Value**	**≤3 months (*n*= 74) Mean ±SD**	**Control group (*n* = 94) Mean ±SD**	***P*-Value**	**>3 months (*n*= 81) Mean ±SD**	**Control group (*n* = 94) Mean ±SD**	***P*-Value**
RR (Ms)	794.16 ± 132.22	865 ± 121	^*^0.001	822.28 ± 126.72	865 ± 121	^*^0.010	806.93 ± 129.25	865 ± 121	^*^<0.0001	847.24 ± 138.30	865 ± 121	0.366
SDNN (Ms)	28.47 ± 28.46	46.50 ± 29.20	^*^<0.0001	42.58 ± 119.92	46.50 ± 29.20	^*^<0.0001	39.84 ± 111.68	46.50 ± 29.20	^*^<0.0001	46.83 ± 133.77	46.50 ± 29.20	^*^<0.0001
RMSSD (Ms)	31.48 ± 37.22	54.90 ± 40.64	^*^<0.0001	35.05 ± 30.59	54.90 ± 40.64	^*^<0.0001	33.61 ± 34.34	54.90 ± 40.64	^*^<0.0001	38.25 ± 35.68	54.90 ± 40.64	^*^0.000
SD1 (Ms)	22.29 ± 26.36	39.89 ± 28.39	^*^<0.0001	24.82 ± 21.66	39.89 ± 28.39	^*^<0.0001	23.80 ± 24.32	39.89 ± 28.39	^*^<0.0001	27.09 ± 25.27	39.89 ± 28.39	^*^0.000
SD2 (Ms)	32.88 ± 31.12	51.52 ± 31.79	^*^<0.0001	35.27 ± 21.96	51.52 ± 31.79	^*^<0.0001	34.27 ± 26.80	51.52 ± 31.79	^*^<0.0001	35.93 ± 23.32	51.52 ± 31.79	^*^<0.0001
SD1/SD2	0.61 ± 0.25	0.76 ± 0.324	^*^0.002	0.66 ± 0.22	0.76 ± 0.324	^*^0.028	0.65 ± 0.24	0.76 ± 0.324	^*^0.000	0.69 ± 0.24	0.76 ± 0.324	0.160
ApEn	1.12 ± 0.15	1.07 ± 0.130	^*^0.023	1.14 ± 0.12	1.07 ± 0.130	^*^0.000	1.14 ± 0.13	1.07 ± 0.130	^*^<0.0001	1.12 ± 0.13	1.07 ± 0.130	^*^0.046
SampEn	1.58 ± 0.37	1.47 ± 0.383	0.066	1.63 ± 0.32	1.47 ± 0.383	^*^0.003	1.62 ± 0.34	1.47 ± 0.383	^*^0.002	1.59 ± 0.32	1.47 ± 0.383	0.059
LF (n.u)	54.39 ± 21.54	44.65 ± 20.71	^*^0.007	48.83 ± 16.71	44.65 ± 20.71	^*^0.006	51.18 ± 19.26	44.65 ± 20.71	^*^0.001	47.29 ± 18.33	44.65 ± 20.71	0.377
HF (n.u)	45.54 ± 21.52	55.28 ± 20.69	^*^0.007	51.04 ± 16.84	55.28 ± 20.69	^*^0.006	48.69 ± 19.23	55.28 ± 20.69	^*^0.001	52.60 ± 18.33	55.28 ± 20.69	0.370
LF/HF	6.33 ± 30.60	1.26 ± 1.42	^*^0.002	1.26 ± 1.15	1.26 ± 1.42	0.099	1.56 ± 1.56	1.26 ± 1.42	^*^0.001	4.22 ± 25.06	1.26 ± 1.42	0.235

*SD, standard deviation; RR, average of RR intervals; N.u, normalized units; SDNN, standard deviation of all normal RR intervals, RMSSD, square root of the mean square of the differences between adjacent normal RR intervals in a time interval; SD1, rapid changes in RR intervals is an SNP index; SD2, long-term changes; SD1/SD2, short-term ratio for long-term range variation; Approximate entropy, ApEn complexity, approximate entropy, regularity of the RR interval series and signal complexity; SampEn, simple entropy, regularity of the RR interval series; LF, low-frequency components, ranging from 0.04 to 0.15 Hz; HF, high-frequency components, ranging from 0.15 to 0.4 HZ; LF/HF, low/high frequency components (normal range 1.5 to 2.0)*.

* *P significant value*.

**Table 5:** Analysis of HRV considering the duration of long COVID-19 and dyspnoea in the control group.

**Variables**	**≤6 months (*n* = 119) Mean ±DP**	**Control group (*n* = 94) Mean ±DP**	***P*-Value**	**>6 months (*n* = 36) Mean ±DP**	**Control group (*n* = 94) Mean ±DP**	***P*-Value**	**Dyspnoea (*n* = 132) Mean ±DP**	**Control group (*n* = 94) Mean ±DP**	***P*-Value**	**Not dyspnoea (*n* = 23) Mean ±DP**	**Control group (*n* = 94) Mean ±DP**	***P*-Value**
RR (Ms)	806.93 ± 129.25	865 ± 121	^*^0.001	830.86 ± 127.96	865 ± 121	0.159	812.97 ± 131.03	865 ± 121	^*^0.002	809.69 ± 118.88	865 ± 121	0.051
SDNN (Ms)	39.84 ± 111.68	46.50 ± 29.20	^*^<0.0001	30.46 ± 19.96	46.50 ± 29.20	^*^0.000	39.81 ± 106.10	46.50 ± 29.20	^*^<0.0001	25.35 ± 20.63	46.50 ± 29.20	^*^<0.0001
RMSSD (Ms)	33.61 ± 34.34	54.90 ± 40.64	^*^<0.0001	34.45 ± 28.44	54.90 ± 40.64	^*^0.002	34.57 ± 33.17	54.90 ± 40.64	^*^<0.0001	29.42 ± 32.35	54.90 ± 40.64	^*^0.000
SD1 (Ms)	23.80 ± 24.32	39.89 ± 28.39	^*^<0.0001	24.40 ± 20.14	39.89 ± 28.39	^*^0.001	24.48 ± 23.48	39.89 ± 28.39	^*^<0.0001	20.82 ± 22.83	39.89 ± 28.39	^*^<0.0001
SD2 (Ms)	34.27 ± 26.80	51.52 ± 31.79	^*^<0.0001	35.00 ± 20.69	51.52 ± 31.79	^*^0.001	35.47 ± 26.30	51.52 ± 31.79	^*^<0.0001	28.52 ± 19.25	51.52 ± 31.79	^*^<0.0001
SD1/SD2	0.65 ± 0.24	0.76 ± 0.324	^*^0.004	0.64 ± 0.20	0.76 ± 0.324	^*^0.050	0.64 ± 0.23	0.76 ± 0.324	^*^0.002	0.67 ± 0.23	0.76 ± 0.324	0.211
ApEn	1.14 ± 0.13	1.07 ± 0.130	^*^<0.0001	1.11 ± 0.15	1.07 ± 0.130	0.213	1.14 ± 0.13	1.07 ± 0.130	^*^<0.0001	1.09 ± 0.16	1.07 ± 0.130	0.501
SampEn	1.62 ± 0.34	1.47 ± 0.383	^*^0.003	1.59 ± 0.34	1.47 ± 0.383	0.111	1.62 ± 0.33	1.47 ± 0.383	^*^0.002	1.55 ± 0.39	1.47 ± 0.383	0.294
LF (n.u)	51.18 ± 19.26	44.65 ± 20.71	^*^0.016	49.39 ± 16.66	44.65 ± 20.71	0.221	50.39 ± 19.02	44.65 ± 20.71	^*^0.031	52.94 ± 16.60	44.65 ± 20.71	0.077
HF (n.u)	48.69 ± 19.23	55.28 ± 20.69	^*^0.015	50.56 ± 16.65	55.28 ± 20.69	0.222	49.50 ± 18.99	55.28 ± 20.69	^*^0.029	46.97 ± 16.58	55.28 ± 20.69	0.076
LF/HF	1.56 ± 1.56	1.26 ± 1.42	^*^0.017	7.90 ±37.51	1.26 ± 1.42	0.063	3.30 ± 19.64	1.26 ± 1.42	^*^0.022	1.46 ± 1.12	1.26 ± 1.42	^*^0.033

*SD, standard deviation; RR, average of RR intervals; N.u, normalized units; SDNN, standard deviation of all normal RR intervals, RMSSD, square root of the mean square of the differences between adjacent normal RR intervals in a time interval; SD1, rapid changes in RR intervals is an SNP index; SD2, long-term changes; SD1/SD2, short-term ratio for long-term range variation; Approximate entropy, ApEn complexity, approximate entropy, regularity of the RR interval series and signal complexity; SampEn, simple entropy, regularity of the RR interval series; LF, low-frequency components, ranging from 0.04 to 0.15 Hz; HF, high-frequency components, ranging from 0.15 to 0.4 HZ; LF/HF, low/high frequency components (normal range 1.5 to 2.0)*.

* *P significant value*.

## Discussion

In this study, patients with long COVID had persistent symptoms of dyspnoea, fatigue, muscle weakness, and chest pain and were mostly women. Long COVID clinical groups with increased sympathetic activity influence of, less parasympathetic activity, and reduced sympathovagal balance were compared. When the participants in the clinical groups with long COVID were compared with the COVID-19-uninfected control group, they demonstrated a decreased sympathovagal balance in the heart. When linear and nonlinear analyses were performed, this population showed changes in HRV, thus suggesting changes in the autonomic control of cardiac function.

The HRV changes observed in the long-term COVID population suggest the need for non-invasive assessments and the early detection of possible changes. The study by Mol et al. (11) demonstrated that higher HRV might predict greater chances of survival in older patients with COVID-19, independent of prognostic factors. Moreover, low HRV predicted ICU admission in the first week after hospitalization. Therefore, HRV measurements may be useful not only for monitoring patients with COVID-19 but also in the early identification of patients with long COVID at risk of clinical deterioration.

The majority of the people infected with SARS-CoV-2 (mild, moderate, or severe) demonstrated chronic signs and symptoms for weeks or months after the infection, lasting 12 weeks or more (19, 20). These signs of potential chronicity were observed in our study of patients who had chronic symptoms for 12 weeks or more.

Carfi et al. (20) reported the occurrence of persistent symptoms (37%) in 179 patients (53 women) for an average of 60 days after the onset of symptoms. Fatigue (53.1%) and dyspnoea (43.4%) persistently occurred in 87.54% of patients with COVID-19. The proportion of females and the prevalence of symptoms were similar to those in our study.

The prevalence of fatigue amongst women in the present study was also confirmed in a study by Kamal et al. (21), which analyzed 287 patients (64.1% women) and reported several persistent manifestations of long COVID and a higher prevalence of fatigue in women (72.8%).

In the present study, those with persistent symptoms were closer to the beginning of COVID-19 recovery, that is, ≤3–6 months of recovery. Al-Aly et al. (22) studied patients with COVID-19, who recovered at least 30 days after their diagnosis for 6 months. This is because the first 30 days or more of the illness after the diagnosis is associated with an increased risk of death; this results from the occurrence of several respiratory, neurological, and cardiovascular disorders, as well as malaise; fatigue; and musculoskeletal pain.

Persistent post-COVID-19 symptoms within 3–6 months after “recovery” from COVID-19 were also described by González-Hermosillo et al. (23), who analyzed 130 patients. Of these, 91.5% reported at least one symptom prior to the onset of infection. The symptom of fatigue persisted among those aged between 40 and 50 years who had long COVID for 3–6 months. As in our study, in the same age group, women were more likely to experience long-term symptoms.

The mechanism of COVID-19 development, immune system response, and Autonomic Nervous System (ANS) are complex subjects. SARS-CoV-2 can activate the innate and adaptive immune responses, generating inflammatory responses that can lead to local and systemic damage (24). Autonomic dysfunction may be mediated by the virus itself. However, during the cytokine storm, vagal stimulation induces an anti-inflammatory response, while sympathetic activation induces the release of pro-inflammatory cytokines. Some studies reported the association between autonomic dysfunction and the short and long-term neurotropism of SARS-CoV-2 (25, 26).

Increase in the influence of sympathetic activity at rest, which can generate an increase in premature deaths, remains of great concern (27). This alteration may increase the HR, while the emergence of cardiovascular diseases predisposes the patient to systemic arterial hypertension and incorrect adaptations of the ANS in response to this (28), thus impairing cardiac regulation.

Heart rate variability has been used to diagnose autonomic regulation, and sympathetic and parasympathetic imbalance occurs in dysautonomia. It is unclear, how dysautonomia with HRV dysregulation occurs in patients with COVID-19 and long COVID. This could be due to neurotropism, hypoxia, and inflammation caused by the autonomic-virus pathway or immune-mediated processes after viral exposure (29). Cardiovascular dysautonomia frequently occurs in patients who recover from COVID-19. There is a reduction in the HRV components (rMSSD and SDNN) when compared with that in uninfected individuals. Despite the scarcity of HRV data, some researchers have been investigating autonomic dysfunction in patients with long COVID to improve disease management and prognosis and limit the progression of the disease (30). Further, the adverse effects of viral infection can generate an increase the sympathetic tone influence, thus preventing the balance in parasympathetic modulation in patients with long COVID.

We reported an increase influence of in the resting sympathetic tone, a decrease in parasympathetic tone, and significant changes in RMSSD and SDNN in long COVID clinical groups compared with those in long-term COVID groups and reduced LF/HF compared with that in the COVID-19-uninfected control group. A previous cross-sectional study conducted by Kaliyaperumal et al. (31) analyzed 106 patients treated for COVID-19 (asymptomatic or mildly to moderately symptomatic). Of these, 63 (59.4%) had COVID-19, while 43 (40.6%) were healthy. The authors demonstrated high rates of autonomic imbalance in patients with COVID-19. Parasympathetic modulation (rMSSD and SDNN) increased in the patients with COVID-19 independent of age, sex, and comorbidities, while the HRV components in LF and HF potencies decreased in COVID-19 patients, when compared with the healthy uninfected individuals.

The parasympathetic activity (RMSSD, SD1, and HF) decreased in long COVID clinical groups; this finding suggests that parasympathetic changes may be associated with mediation of the inflammatory process. However, in a meta-analysis of 159 studies by Williams et al. (32), a negative association was found between HRV and vagal indices (e.g. HF), SDNN, and inflammation markers. SDNN was strongly associated with inflammatory markers and had greater effects in women than in men.

Other decreases in parasympathetic modulation were reported by Gifford et al. (33), who examined the autonomic function and HRV after extreme resistance exercise. They reported that healthy women have a lower sympathetic profile; an increase in HRV within 15 days after performing exercise showed better parasympathetic activity (RMSSD, SD1, and HF), increased global HRV (SD1/SD2), and increased SampEn.

In the present study, changes in ApEn and SampEn entropies were not observed in the long COVID clinical groups compared with that in the COVID-19-free control group. However, Bajic, Ðajić, and Milovanović (34), when analyzing the different entropies (Apen, SampEn, binary, sample, and multiscale) of 116 patients with COVID-19 (mild to severe) and 77 healthy controls, only found significant cross-entropies in heart rate signals and systolic pressure. Most of the patients with COVID-19 had lower SampEn values compared with those in the control group. Considering that ANS dysfunctions are associated with COVID-19 severity, we believe that signal acquisition is complex; moreover, no difference was found in the entropies between patients with COVID-19 and controls.

Heart rate variability has been assessed in other studies to determine autonomic functions (35). Linear and nonlinear methods were used to analyse HRV to assess cardiac modulation (36). Our study demonstrated HRV alterations in the long COVID population with cardiac autonomic dysfunction; increased influence of sympathetic activity at rest was associated with increased HR and blood pressure levels, cardiovascular problems, poorer prognosis, and sudden death. However, this finding still needs to be extensively explored to understand the mechanisms leading to these alterations. In addition, the usefulness of this tool in clinical practice should be evaluated.

### Strengths and Limitations

Heart rate variability analysis was performed using a cardiofrequency meter, which is influenced by individual and behavioral factors. The study was performed at a single center and had a small sample size. The sample was representative of the population studied; few studies described in the scientific literature used HRV analysis for assessing cardiac modulation in the long COVID population. However, further studies need to be conducted to understand the repercussions of long COVID in different body organs and on breathing controls to understand whether long COVID impacts cardiac autonomic modulation. The results collected in this study will be fundamental for the initial understanding of cardiac autonomic alterations in patients with long COVID.

## Conclusions

Our results demonstrated that the long COVID clinical groups showed reduced HRV compared with the COVID-19-uninfected control group. Short-term linear and nonlinear methods demonstrated good precision in this population. Therefore, changes in long COVID should be monitored to understand its involvement in cardiac autonomic modulation and detect possible cardiovascular changes for short- or long-term prevention. In particular, increased influence of sympathetic activity may be linked with cardiovascular imbalances, chronic disease, and sudden death. Hence, further tests and clinical trials should be conducted to understand the after-effects of long COVID on cardiac autonomous modulation. Although changes in the ANS were observed, it is unclear, whether the changes were caused directly or indirectly by infection or systemic inflammatory state in patients who recovered from COVID-19.

## Data Availability Statement

The original contributions presented in the study are included in the article/supplementary material, further inquiries can be directed to the corresponding author/s.

## Ethics Statement

The studies involving human participants were reviewed and approved by Research Ethics Committee of the Pará State University (UEPA) opinion number 4.252.664. The patients/participants provided their written informed consent to participate in this study.

## Author Contributions

JQ and LF: project administration, support, supervision, review, and scientific collaboration. MS and RR: support, review, and edition. CS and ST: investigation, data collection, and written. KM: investigation, data collection, written, and edition and review. All authors read and approved the final manuscript.

## Funding

Funding was provided through the Amazonia Paraense of Support Foundation to Research (FAPESPA) 006/2020, Coordination for the Improvement of Higher Education Personnel (CAPES), and Secretariat for Science, Technology and Higher, Professional and Technological Education (SECTET).

## Conflict of Interest

The authors declare that the research was conducted in the absence of any commercial or financial relationships that could be construed as a potential conflict of interest.

## Publisher's Note

All claims expressed in this article are solely those of the authors and do not necessarily represent those of their affiliated organizations, or those of the publisher, the editors and the reviewers. Any product that may be evaluated in this article, or claim that may be made by its manufacturer, is not guaranteed or endorsed by the publisher.

## References

[1] Hamid S Mir MY Rohela GK. Novel coronavirus disease (COVID-19): a pandemic (epidemiology, pathogenesis and potential therapeutics). New Microbes New. (2020) 35:100679. 10.1016/j.nmni.2020.100679 32322401 PMC7171518

[2] Ministry of Health (BR). Special Epidemiological Bulletin. Coronavirus Panel-Covid-19. (2021). https://covid.saude.gov.br/. Accessed October 4, 2021.

[3] Sher L. Post-COVID syndrome and suicide risk. QJM. (2021) 114:95–8. 10.1093/qjmed/hcab007 33486531 PMC7928695

[4] Kingstone T Taylor AK O'donnel CA Atherton H Blane DN Chew-Graham CA. Finding the ‘right’ GP: a qualitative study of the experiences of people with long-COVID. BJGP Open. (2020) 4:1–12. 10.3399/bjgpopen20X101143 33051223 PMC7880173

[5] Mascia G Pescetelli F Baldari A Gatto P Seitun S Sartori P et al. Interpretation of elevated high-sensitivity cardiac troponin I in elite soccer players previously infected by severe acute respiratory syndrome coronavirus 2. Int J Cardiol. (2021) 326:248–51. 10.1016/j.ijcard.2020.11.039 33242510 PMC7682525

[6] Li DL Davogustto G Soslow JH Wassenaar JW Parikh AP Chew JD et al. Characteristics of COVID-19 myocarditis with and without multisystem inflammatory syndrome. Am J Cardiol. (2022) 168:135–41. 10.1016/j.amjcard.2021.12.031 35058054 PMC8767902

[7] Becker RC. Autonomic dysfunction in SARS-COV-2 infection acute and long-term implications COVID-19 editor's page series. J Thromb Thombolysis. (2021) 17:1–16. 10.1007/s11239-021-02549-6 34403043 PMC8367772

[8] Berger M Raffin J Pichot V Hupin D Garet M Labeix P et al. Effect of exercise training on heart rate variability in patients with obstructive sleep apnea: a randomized controlled trial. Scand J Med Sci Sports. (2019) 29:1254–62. 10.1111/sms.13447 31050034

[9] Francesco B Grazia BM Emanuele G Valentina F Sara C Chiara F et al. Linear and nonlinear heart rate variability indexes in clinical practice. Comput Math Methods Med. (2012) 2012:1–5. 10.1155/2012/219080 22400047 PMC3287009

[10] Task Force of the European Society of Cardiology and the North American Society of Pacing and Electrophysiology. Eur Heart J. (1996) 17: 354–381. 8737210

[11] Mol MBA Strouss MTA Osch FHMV Vogelaar FJ Barten DG Farchi M. Heart-rate-variability (HRV), predicts outcomes in COVID-19. PLoS ONE. (2021) 16:e0258841. 10.1371/journal.pone.0258841 34710127 PMC8553073

[12] Arêas GPT Caruso FCR Simões RP Simões VC Jaenisch RB Sato TO. Ultra-short-term heart rate variability during resistance exercise in the elderly. Braz J Med Biol Res. (2018) 51:e6962. 10.1590/1414-431x20186962 29791599 PMC6002140

[13] Burr RL. Interpretation of normalized spectral heart rate variability indices in sleep research: a critical review. Sleep-New York Then Westchester. (2007) 30:913–9. 10.1093/sleep/30.7.913 17682663 PMC1978375

[14] Vanderlei LC Pastre CM Hoshi RA Carvalho TD Godoy MF. Noções Básicas de variabilidade da frequência cardíaca e sua aplicabilidade clínica. Rev Bras Cir Cardiovasc. (2009) 24:205–17. 10.1590/S0102-76382009000200018 19768301

[15] Martin J Schneider F Kowalewskij A Jordan D Hapfelmeier A Kochs EF. Linear and non-linear heart rate metrics for the assessment of anaesthetists workload during general anaesthesia. Br J Anaesth. (2016) 117:767–74. 10.1093/bja/aew342 27956675

[16] Hoshi RA Pastre CM Vanderlei LC Godoy MF. Poincaré plot indexes of heart rate variability: Relationships with other nonlinear variables. Auton Neurosc. (2013) 177:271–4. 10.1016/j.autneu.2013.05.004 23755947

[17] Pincus S. Approximate entropy (ApEn) as a complexity measure. Chaos. (1995) 5:110–7. 10.1063/1.166092 12780163

[18] Thu TNP Hérnandez AL Costet N Patural H Pichot V Carrault G et al. Improving methodology in hearth rate variability analysis for the premature infants: impact of the time length. PLoS ONE. (2019) 14:1–14. 10.1371/journal.pone.0220692 31398196 PMC6688831

[19] Carod-Artal FJ. [Post-COVID-19 syndrome: epidemiology, diagnostic criteria and pathogenic mechanisms involved]. Rev Neurol. (2021) 72:384–96. 10.33588/rn.7211.2021230 34042167

[20] Carfi A Bernabei R Landi F. Persistent symptoms in patients after acute COVID-19. JAMA. (2020) 324:603–5. 10.1001/jama.2020.12603 32644129 PMC7349096

[21] Kamal M Omirah MA Hussein A Saeed H. Assessment and characterization of Post-COVID-19 manifestations. Int J Clin Pract. (2021) 75:e13746. 10.1111/ijcp.13746 32991035 PMC7536922

[22] Al-Aly Z Xie Y Bowe B. High-dimensional characterization of post-acute sequelae of COVID-19. Nature. (2021) 594:259–64. 10.1038/s41586-021-03553-9 33887749

[23] González-Hermosillo JA Martínez-López JP Carrillo-Lampón SA Ruiz-Ojeda D Herrera-Ramírez S Amezcua-Guerra LM. Post-acute COVID-19 symptoms, a potential link with myalgic encephalomyelitis/chronic fatigue syndrome: a 6-month survey in a Mexican cohort. Brain Sci. (2021) 11:1–13. 10.3390/brainsci11060760 34201087 PMC8227652

[24] Anka AU Tahir MI Abubakar SD Alsabbagh M Zian Z Hamedifar H. Coronavirus disease 2019 (COVID-19): an overview of the immunopathology, serological diagnosis and management. Scand J Immunol. (2020) 93:e12998. 10.1111/sji.12998 33190302 PMC7744910

[25] Dani M Dirksen A Taraborreli P Torocastro M Panagopoulos D Sutton R. Autonomic dysfunction in ‘long COVID’: rationale, physiology and management strategies. Clin Med. (2021) 21:e63–7. 10.7861/clinmed.2020-0896 33243837 PMC7850225

[26] Hu J Jolkkoken J Zhao C. Neurotropism of SARS-CoV-2 and its neuropathological alterations: similarities with other coronaviruses. Neurosci Biobehav Rev. (2020) 119:184–93. 10.1016/j.neubiorev.2020.10.012 33091416 PMC7571477

[27] Notarius CF Millar PJ Floras JS. Muscle sympathetic activity in resting and exercising humans with and without heart failure. Appl Physiol Nutr Metab. (2015) 40:1107–15. 10.1139/apnm-2015-0289 26481289

[28] Júnior JRZ Viana AO. de Melo Gel, de Angelis K. The impact of sedentarism on heart rate variability (HRV) at rest and in response to mental stress in young women. Physiol Rep. (2018) 6:1–8. 10.14814/phy2.13873 30238692 PMC6148327

[29] Barizien N Guen ML Russel S Touche P Huang F. Vallée. Clinical characterization of dysautonomia in long COVID-19 patients. Sci Rep. (2021) 11:14042. 10.1038/s41598-021-93546-5 34234251 PMC8263555

[30] Shah B Kunal S Bansal A Jain J Poundrik S Shetty MK. Heart rate variability as a marker of cardiovascular dysautonomia in post-COVID-19 syndrome using artificial intelligence. Indian Pacing Electrophysiol J. (2022) 22:70–6. 10.1016/j.ipej.2022.01.004 35101582 PMC8800539

[31] Kaliyaperumal D. Karthikeyan Rk, Alagesan M, Ramalingam S. Characterization of cardiac autonomic function in COVID-19 using heart rate variability: a hospital based preliminar observational study. J Basic Clin Physiol Pharmacol. (2021) 32:247–53. 10.1515/jbcpp-2020-0378 33705614

[32] Williams DP Koening J Carnevali L Sgoifo A Jarcok MN Sternberg EM Thayer JF. Heart rate variability and inflammation: a meta-analysis of human studies. Brain Behav Immun. (2019) 80:219–26. 10.1016/j.bbi.2019.03.009 30872091

[33] Gifford RM Boos CJ Reynolds RM Woods DR. Recovery time and heart rate variability following extreme endurance exercise in healthy women. Physiol Rep. (2018) 6:e13905. 10.14814/phy2.13905 30381902 PMC6209688

[34] Bajić D ðajić V Milovanović B. Entropy analysis of COVID-19 cardiovascular signals. Entropy. (2021) 23:87. 10.3390/e23010087 33435378 PMC7826611

[35] Hayano J Yuda E. Pitfalls of assessment of autonomic function by heart rate variability. J Physiol Anthopol. (2019) 38:1–8. 10.1186/s40101-019-0193-2 30867063 PMC6416928

[36] Hoshi RA Andrea RY Santos IS Dantas EM Mill JG Lotufo PA. Linear and nonlinear analyses of heart rate variability following orthostatism in subclinical hypothyroidism. Medicine. (2019) 98:1–7. 10.1097/MD.0000000000014140 30681577 PMC6358401

